# Sustainable microalgae-based Docosahexaenoic Acid–Silver nanoparticles for biomedical applications

**DOI:** 10.3389/fbioe.2026.1830568

**Published:** 2026-05-28

**Authors:** Ashfaq Ahmad, Kaumeel Chokshi, Dalaver Anjum, Peter R. Corridon, Abdelmoneim H. Ali, Mayssa Hachem

**Affiliations:** 1 Department of Biological Sciences, College of Medicine and Health Sciences, Khalifa University of Science and Technology, Abu Dhabi, United Arab Emirates; 2 Department of Chemistry, College of Engineering and Physical Sciences, Khalifa University of Science and Technology, Abu Dhabi, United Arab Emirates; 3 Food Security and Technology Center, Khalifa University of Science and Technology, Abu Dhabi, United Arab Emirates; 4 Department of Physics, Khalifa University of Science and Technology, Abu Dhabi, United Arab Emirates; 5 Department of Biomedical Engineering and Biotechnology, College of Medicine and Health Sciences, Khalifa University of Science and Technology, Abu Dhabi, United Arab Emirates; 6 Department of Food Science, College of Agriculture and Veterinary Medicine, United Arab Emirates University (UAEU), Al Ain, United Arab Emirates

**Keywords:** antimicrobial applications, biomedical drug delivery, DHA-loaded AgNPs, docosahexaenoic acid (DHA), sustainable nanotechnology

## Abstract

**Introduction:**

Docosahexaenoic acid (DHA) is an essential omega-3 polyunsaturated fatty acid with well-established health benefits. However, its poor water solubility and high susceptibility to oxidation limit its application in the biomedical field. In this study, DHA was extracted and purified from the marine microalga *Aurantiochytrium limacinum* and incorporated into silver nanoparticles (AgNPs) to increase their stability for use in biomedical applications.

**Results:**

Microscopic characterization confirmed the formation of nanosized, predominantly spherical AgNPs with an effective surface association with DHA. SEM and TEM analyses demonstrated distinct morphological changes following DHA loading, whereas XRD revealed reduced crystallinity, indicating surface modification. FTIR spectroscopy further confirmed the interactions between the DHA functional groups and the AgNP surface.

**Discussion:**

This study demonstrates a sustainable approach for producing microalgae-derived DHA-loaded AgNPs with enhanced structural stability and multifunctional properties. DHA-AgNPs, developed for biomedical applications, combine antimicrobial activity with improved DHA delivery and support the development of green nanomedicines.

## 1 Introduction

Docosahexaenoic acid (DHA; 22:6 n-3) is a long-chain omega-3 polyunsaturated fatty acid that plays an essential role in human health, particularly in neural development, vision, and cardiovascular protection (Hachem et al., 2020; Hachem and Nacir, 2022). Although fish oil is a conventional source of DHA, several factors, including marine pollution, overfishing, and the oxidative instability of fish-derived lipids, have necessitated the exploration of alternative and sustainable microbial sources (Liu et al., 2025). Microalgae are a valuable source of proteins, n-3 polyunsaturated fatty acids (PUFA), polysaccharides, vitamins, pigments, and antioxidants. In particular, n-3 polyunsaturated fatty acids have gained attention because of their nutritional and physiological properties and health benefits, including antioxidant, antihypertensive, anti-inflammatory, immune regulatory, antiviral, hepatoprotective, neuroprotective, cardiovascular protective, cholesterol-reduction, and anticancer effects (Ahmad and Syed, 2023; Sarıtaş et al., 2024; Siddhnath et al., 2024; Ahmad and Ashraf, 2025; Prates, 2025). A heterotrophic marine microalga from the Thraustochytrid family, *Aurantiochytrium limacinum*, has garnered significant attention because of its remarkable capacity for lipid accumulation, with DHA comprising 40%–50% of its total fatty acids (Li et al., 2015; Aini et al., 2022). However, the poor solubility of DHA in water and its susceptibility to oxidation limit its applications. To overcome these limitations, DHA can be encapsulated in nanoparticles, which enhances its stability and bioavailability (Lv and Xu, 2022; Ali et al., 2024; Chokshi et al., 2026). Silver nanoparticles (AgNPs) are among the most versatile inorganic platforms in biomedicine, offering broad antimicrobial activity, antibiofilm efficacy, and tunable optical and electrical properties that enable their use in sensing and therapy. The field is advancing toward sustainable synthesis methods and safer-by-design surfaces to address the translational challenges of the cytotoxicity and environmental impact of nanomaterials. These developments enable hybrid systems to integrate the antimicrobial capabilities of AgNPs with bioactive molecules that modulate host response (Rodrigues et al., 2024). AgNPs have been extensively used in medicine. Their efficacy is primarily due to their nanoscale dimensions, which confer increased surface area-to-volume ratios (Yin et al., 2020; Ali et al., 2024).

Recent advances in nanotechnology have led to the widespread application of AgNPs owing to their antibacterial, antifungal, antiviral, anti-inflammatory, and anticancer activities. The diverse characteristics of AgNPs enable their use in biological, biomedical, and cosmetic applications. In nanomedicine, silver nanoparticles (AgNPs) have emerged as versatile platforms for drug delivery owing to their high surface area-to-volume ratio, which enables efficient drug loading and surface functionalization. Their favorable toxicity profiles, cost efficiency, and biocompatibility further support their integration into therapeutic nanosystems. Importantly, the simplicity of solid-state synthesis and extraction offers a scalable route for clinical translation, while allowing precise control over nanoparticle size, morphology, and dispersion—parameters that critically influence biodistribution, cellular uptake, and therapeutic efficacy in nanomedicine applications (Ali et al., 2024; Das et al., 2025). AgNPs exhibit potent antimicrobial and antibiofilm activities against a wide range of pathogens, including bacteria and fungi. These effects are attributed to their ability to disrupt microbial membranes and inhibit biofilm formation (Kavipriya et al., 2024; Lin et al., 2024; Lakkakula et al., 2026). Microalgae-derived AgNPs have demonstrated strong antibacterial activity, with minimum inhibitory concentrations as low as 5 μg/mL for pathogens such as *Escherichia coli* (Juliana et al., Lakkakula et al., 2026). However, DHA’s high degree of unsaturation renders it particularly sensitive to oxidative degradation, limiting its shelf life and biological activity. To address these issues, nanotechnology-based delivery systems have been developed to entrap DHA into protective matrices at the nanoscale level (Lv and Xu, 2022; Ali et al., 2024). Nanoparticle formulations, such as liposomes, nanoemulsions, solid lipid nanoparticles (SLNs), and polymeric nanoparticles, can enhance DHA’s oxidative stability, improve its aqueous dispersibility, and facilitate targeted or controlled release in biological systems (Das et al., 2025; Otaegui et al., 2025). The production of DHA nanoparticles using oils derived from *A. limacinum* represents the integration of microbial biotechnology and advanced nanodelivery systems. This technique would not only protect DHA from environmental stressors but also be key to sustainable production. In addition, *A. limacinum* can be grown on inexpensive substrates under controlled conditions, thus providing large-scale production feasibility, both economically and environmentally (Li et al., 2015; Olsen et al., 2023). Recently, fermentation and extraction processes have been optimized to enhance the lipid yields and encapsulation efficiency of nanoparticles, further underlining the potential of this organism as a commercial biofactory for high-value omega-3 delivery systems (Ali et al., 2024; Lin et al., 2025).

DHA nanoparticles derived from *A. limacinum* represent a significant advancement in the formulation of functional foods and nutraceuticals, offering enhanced stability, improved bioavailability, and a sustainable alternative to fish-based omega-3 products, which aligns with current trends in green biotechnology and health-oriented nanoscience. In this study, we report the preparation and characterization of nanoparticles formulated using DHA purified from *A. limacinum* for biomedical applications.

## 2 Materials and methods

### 2.1 Cultivation of *Aurantiochytrium limacinum*

*A. limacinum* ATCC MYA-1381 cells were obtained from the American Type Culture Collection (ATCC). The cells were grown in 1 L Erlenmeyer flasks containing 500 mL of ATCC-790 medium supplied with 100 mg/L ampicillin, kanamycin, and streptomycin (Dirir et al., 2025). Cell cultivation was performed in eight flasks to obtain sufficient biomass for the experiment. Each culture was incubated at 150 rpm in a shaker at 25 °C until the cells reached the stationary phase. Cell growth was monitored by measuring OD_660_. After cultivation, cells were harvested by centrifugation at 450 *g* for 10 min at 4 °C and washed twice with distilled water. The collected cell pellet was frozen at −80 °C, lyophilized, and the obtained powder was stored at 4 °C.

### 2.2 Lipid extraction

Lyophilized biomass was used for lipid extraction as previously described (Dirir et al., 2025). A chloroform/methanol mixture (CHCl_3_/CH_3_OH 2:1, v/v) was added to a known quantity (500 mg) of biomass (Folch et al., 1957). Vitamin E (500 μg per g biomass) was incorporated into the extraction mixture to prevent lipid oxidation. The samples were sonicated for 5 min under cold conditions and incubated overnight at 4 °C. After incubation, the mixture was centrifuged at 450 *g* for 10 min at 4 °C, and the supernatant was collected. The extraction process was repeated twice under identical conditions, and all lipid extracts were pooled. The solvent was evaporated using a nitrogen stream, and the resulting dried lipid samples were stored at −20 °C until further analysis.

### 2.3 Production of fatty acid methyl esters

To determine the fatty acid composition, fatty acid methyl esters (FAMEs) were prepared for Gas Chromatography Flame Ionization Detector (GC-FID) analysis (Iverson and Sheppard, 1975). FAMEs were produced using a modified AOAC 969.33 method with toluene/methanol (2:3, v/v) and BF_3_/methanol (14%), where BF_3_ is a catalyst for methylation and metanalysis (Ackman, 1998). The reaction mixture was heated at 100 °C for 90 min, after which the tubes were immediately cooled in an ice bath. Subsequently, 10% potassium carbonate (K_2_CO_3_) and isooctane were added to the reaction mixture. The samples were centrifuged at 450 *g* for 5 min at 4 °C, and the upper organic phase containing the FAMEs was carefully collected. The solvent was then evaporated under a nitrogen stream, and the resulting FAMEs were stored at −20 °C until further analysis.

### 2.4 Gas chromatography-flame ionization detection (GC-FID) analysis

Gas chromatographic analysis of the FAMEs was performed as described by Dirir et al. (2025) using an Agilent 7890B GC system equipped with a flame ionization detector (FID) and an Rt-2560 capillary column (100 m × 0.25 mm × 0.20 µm; Restek, United States) (Dirir et al., 2025). Helium was used as the carrier gas at a constant flow rate of 2 mL/min. The FID temperature was maintained at 280 °C, with gas flow rates of 350, 35, and 30 mL/min for synthetic air, hydrogen, and helium, respectively. Concentrated FAMEs were dissolved in 1 mL of hexane prior to analysis, and a 1 µL aliquot was injected into the gas chromatography (GC) system. A series of blanks (hexane, HPLC grade) and a 37 FAME standard mix (Restek, United States) were analyzed alongside the samples to enable the qualitative and quantitative identification of the individual fatty acids. The oven temperature program was as follows: the initial temperature was set at 80 °C and held for 10 min, then increased at 7 °C/min to 170 °C and maintained for 10 min. The temperature was subsequently increased at 12 °C/min to 205 °C, held for 20 min, followed by an increase of 20 °C/min to 220 °C and held for 15 min. Finally, the temperature was increased to 230 °C at a rate of 15 °C/min and maintained for 20 min at that temperature.

### 2.5 Purification of DHA methyl ester (DHA-ME) using HPLC

An Agilent Infinity 1260 HPLC model was used to purify methylated DHA (DHA-ME) from the total FAMEs. A Cadenza CD-C18 HT (4.6 mm × 150 mm, 3 μm) column and a mobile phase consisting of acetonitrile/water (80/20, v/v) in isocratic mode with a flow rate of 1 mL/min were used for the analysis. The injection volume was 20 μL. The wavelength for UV detection was set to 205 nm. DHA-ME was collected at 43.53 min, and the solvent was evaporated using a rotary evaporator to obtain DHA-ME.

### 2.6 Alkaline hydrolysis of DHA-ME

DHA-ME (10 mg) was hydrolyzed by adding 0.6 mL of potassium hydroxide/methanol (KOH/CH_3_OH; 9%) and incubated at 40 °C for 30 min. The medium was then acidified to pH 3 with concentrated acetic acid (CH_3_COOH), and DHA was extracted using 2.8 mL of a chloroform/methanol mixture (2:1, v/v). After mixing and centrifugation (450 × g for 10 min at 40 °C), the organic phase (bottom phase) was recovered and evaporated under nitrogen atmosphere.

### 2.7 Preparation of docosahexaenoic acid-loaded silver nanoparticles (DHA-AgNPs)

Silver nanoparticles (AgNPs) were synthesized using a solid-state approach (Das et al., 2025). Initially, 2 g of cellulose nanocrystals (CNC) were ground with 0.4 g of sodium hydroxide (NaOH) using a mortar and pestle for 10 min to obtain a fine powder mixture. Subsequently, 0.5 g of silver nitrate (AgNO_3_) was added, and grinding continued until a yellow color indicated the formation of AgNPs. The resulting powder was calcined at 400 °C for 2 h to eliminate residual or unreacted substances. The synthesized AgNPs (0.5 g) were dispersed in 100 mL of water containing Tween-40 as a surfactant. To load DHA, 8 mg of DHA was gradually added to the AgNP suspension until a uniform solution was achieved. The mixture was ultrasonicated for 20 min and ultracentrifuged at 20,000 rpm for 2 h. The final DHA-AgNP product was dried at 50 °C and stored for subsequent analysis and application.

### 2.8 DHA-nanoparticles characterization

X-ray diffraction (XRD) analysis was performed using a Panalytical RAYONS-X XRD spectrometer (Japan) to identify and characterize the crystalline phases of AgNPs. Diffraction patterns were recorded over a 2θ range of 30°–80°. Fourier-transform infrared (FTIR) spectroscopy (Shimadzu FTIR-8400S, Japan) was used to verify the presence of AgNPs in the samples. Spectral data were collected in the range of 4,000–500 cm^-1^. Field-emission scanning electron microscopy (FE-SEM; JEOL JSM-7610F FEG-SEM) was used to examine the surface morphology and microstructures of both pure and DHA-loaded AgNPs. The morphology of the DHA–AgNPs, including the particle size and shape, was characterized using a Talos 200 transmission electron microscope (Thermo Fisher Scientific) operated at 200 kV. Bright-field TEM was used to assess the overall morphology, whereas high-resolution TEM (HRTEM) enabled a detailed structural analysis.

## 3 Results and discussion

### 3.1 Biomass production from *A. limacinum*

*A. limacinum* ATCC MYA-1381 cells were harvested once the culture reached the stationary phase (OD_660_ ∼ 1.5). After 10 days of cultivation, a total of 4 g of dry biomass was obtained from 4 L of culture, corresponding to a dry biomass productivity of 100 mg/L/day.

### 3.2 Lipid extraction and FAME profile by GC-FID analysis

**FIGURE 1:**
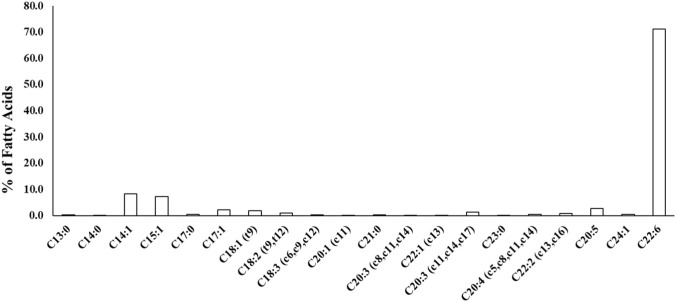
Fatty acid composition of lipids extracted from *Aurantiochytrium limacinum* ATCC MYA-1381 using GC-FID analysis.

1.06 g of total lipid was recovered from 3 g of the lyophilized biomass used for the lipid extraction. After the transesterification reaction, 563 mg of FAMEs were obtained, which were dissolved in 1 mL of hexane, and 1 µL aliquots were injected into the GC-FID system for analysis. Gas chromatography revealed 20 distinct fatty acids (Figure 1). Among these, docosahexaenoic acid (DHA, C22:6n-3) was the most abundant fatty acid. 71.1% of the total FAMEs, respectively. Myristoleic acid (C14:1) and pentadecenoic acid (C15:1) were detected at 8.36% and 7.28% levels, respectively. The remaining fatty acids were present at relatively low concentrations in the oil.

### 3.3 HPLC purification

**FIGURE 2:**
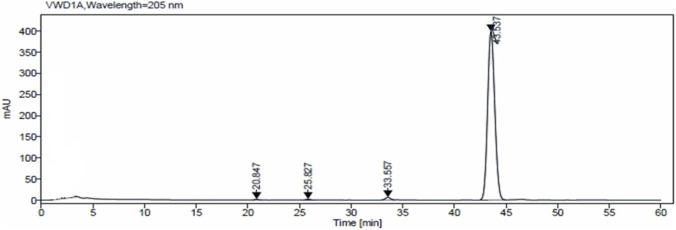
HPLC chromatogram of FAME produced by microalgae.

Figure 2 shows the HPLC chromatogram of FAME in the reverse-phase mode. DHA-ME was eluted and collected at 43.53 min. The solvent was evaporated from the collected fractions (50 fractions), and DHA-ME was quantified. The amount of DHA-ME (10 mg) was recovered and stored at −20 °C until hydrolysis was performed.

### 3.4 Characterization of AgNPs and DHA-Loaded AgNPs

**FIGURE 3:**
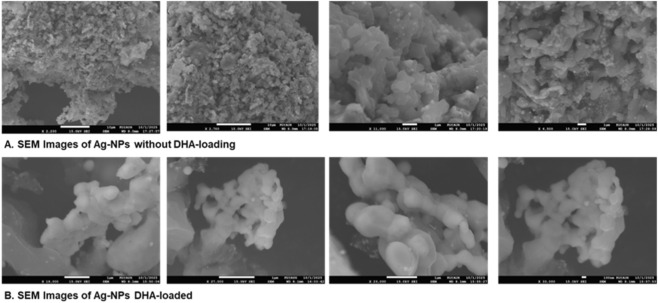
SEM micrographs of AgNPs: **(A)** without DHA loading; **(B)** with DHA-loading.

Hydrolysis of the recovered DHA-ME (10 mg) resulted in approximately 8 mg of free DHA, which was subsequently used to formulate DHA-loaded AgNPs. Scanning electron microscopy (SEM) was used to investigate the surface morphology and structural characteristics of the AgNPs synthesized on cellulose before and after DHA loading. SEM micrographs of the unmodified AgNPs (Figure 3A) revealed a relatively uniform distribution of spherical nanoparticles with minimal agglomeration. The particles were well dispersed across the cellulose matrix, indicating effective synthesis and particle stabilization. Notable morphological changes were observed after DHA loading (Figure 3B). The surfaces of the DHA-loaded AgNPs exhibited increased aggregation and a more irregular structure. This transformation suggests a successful interaction between DHA molecules and AgNPs, likely owing to surface adsorption or encapsulation. The presence of DHA may have influenced particle–particle interactions, leading to clustering and altered surface textures. These morphological differences confirm the incorporation of DHA into the AgNP system and may have implications for the functional properties of the nanoparticles, such as their bioavailability, stability, and potential biomedical applications. The increased surface roughness and aggregation of the DHA-loaded samples could enhance cellular uptake or controlled release, depending on the intended application (Ali et al., 2024; Do et al., 2025).

**FIGURE 4:**
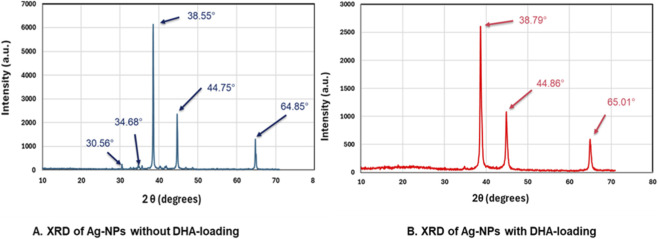
XRD patterns of AgNPs: **(A)** without DHA-loading; **(B)** with DHA-loading.

Figure 4 shows the XRD patterns of AgNPs before and after DHA loading. The diffraction peaks observed for the unmodified AgNPs (Figure 4A) at 2θ values of approximately 30.56°, 34.68°, 38.55°, 44.75°, and 64.85° correspond to the characteristic planes of face-centered cubic (FCC) silver, confirming the successful synthesis of crystalline AgNPs. The sharp and intense peaks indicate a high degree of crystallinity in these samples. In contrast, the XRD pattern of the DHA-loaded AgNPs (Figure 4B) shows fewer and less intense peaks, primarily at 38.79°, 44.86°, and 65.01°. The reduction in peak intensity and number suggests partial masking or alteration of the crystalline structure due to DHA incorporation. This may be attributed to the surface interactions between DHA molecules and AgNPs, which could lead to reduced crystallinity or the formation of an amorphous layer around the nanoparticles. These findings support the successful loading of DHA onto AgNPs and suggest that structural modifications may affect the functional properties of nanoparticles, including drug release behavior, stability, and biological activity (Hussein et al., 2019; Ali et al., 2024).

**FIGURE 5:**
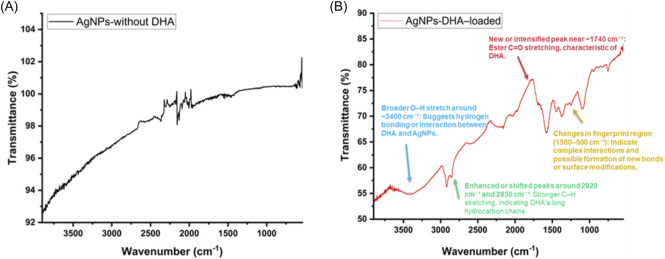
FTIR spectra of AgNPs: **(A)** without DHA; **(B)** with DHA loading.

Figure 5 shows the FTIR spectra of AgNPs before and after DHA loading, providing insights into the chemical interactions and functional groups present in the AgNPs. The spectrum of the unmodified AgNPs (Figure 5A) shows a relatively smooth transmittance curve with minimal distinct peaks, indicating the absence of complex organic functional groups in their structure. This confirmed the purity of the synthesized AgNPs and the absence of surface-bound organic molecules in the sample. In contrast, the FTIR spectrum of the DHA-loaded AgNPs (Figure 5B) showed several significant changes. The prominent peak near 1740 cm^−1^ corresponds to the ester C=O stretching, a characteristic functional group of DHA, confirming the successful incorporation of DHA into the polymer. Additionally, a broad absorption band at approximately 3,400 cm^1^ indicates O–H stretching, suggesting hydrogen bonding interactions between DHA and the nanoparticle surface. Further peaks at 2,920 cm^-1^ and 2,850 cm^-1^ are attributed to C–H stretching vibrations, which are consistent with the long hydrocarbon chains of DHA. Modifications in the fingerprint region (1,500–500 cm^-1^) suggest structural changes and the possible formation of new bonds, indicating the effective surface functionalization of the AgNPs by DHA. These spectral differences validated the presence of DHA on the nanoparticle surface and suggested strong molecular interactions, which may enhance the stability, bioactivity, and potential biomedical applications of DHA-loaded AgNPs (Kumari et al., 2016; Do et al., 2025).

Although DHA-AgNPs offer significant therapeutic potential, their toxicity must be carefully considered. Further research is needed to optimize their efficacy, understand their mechanisms of action, and mitigate potential health risks associated with their use. In conclusion, DHA-loaded AgNPs represent a promising approach to enhance DHA’s therapeutic benefits of DHA while leveraging the unique properties of silver nanoparticles. This combination could lead to more effective treatments for a range of diseases, provided that their safety and efficacy are thoroughly evaluated in future studies.

**FIGURE 6:**
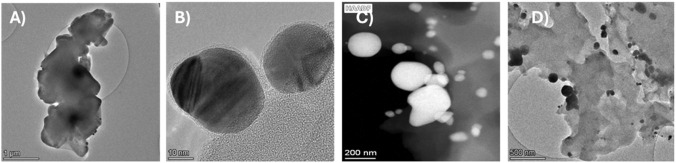
TEM images of DHA-loaded AgNPs at different scale bars magnification: **(A)** 1 µm; **(B)** 10 nm; **(C)** 200 nm; **(D)** 500 nm.

Transmission electron microscopy (TEM) was performed to examine the morphology and structural features of the DHA-loaded AgNPs (Figures 6A–D). At the lowest magnification (Figure 6A; 1 µm scale bar), larger aggregated or flake-like structures containing darker electron-dense regions were observed. These dark spots likely correspond to clustered AgNPs that are entrapped within the DHA-rich organic matrix. The large-scale morphology suggests that localized agglomeration occurred during sample drying or preparation of the TEM grids. However, the absence of complete particle fusion indicates that the nanoparticles retained their structural integrity. At the highest magnification (Figure BA; 10 nm scale bar), the nanoparticles appeared relatively spherical to quasi-spherical with smooth and well-defined edges. The nanoparticles exhibited electron-dense dark cores, characteristic of metallic silver. This confirmed the successful formation of the nanoparticles. A faint, lighter peripheral region surrounding some particles was also observed, which could be a result of DHA adsorption or encapsulation on the nanoparticle surface. It is likely that this coating functions as a stabilizing shell, thereby preventing direct contact between particles and reducing particle aggregation. In this image, the relatively narrow size distribution suggests a controlled nucleation and growth process.

As depicted in Figure 6C, at a magnification level of 200 nm, multiple nanoparticles exhibiting spherical to oval morphologies were dispersed across the field. Although some particles appeared in close proximity or as small clusters, their boundaries remained distinct, indicating physical aggregation rather than an irreversible fusion. The differences in the contrast between the particles may be due to differences in the particle thickness or orientation during imaging. The image also confirms that the nanoparticles remained within the nanoscale range after loading with DHA. Nanoparticles can be seen embedded within or associated with a lighter amorphous matrix-like structure in Figure 6D (500 nm scale bar), which may be excess DHA, residual biomolecules, or an organic capping agent. Small, dark, electron-dense AgNPs were distributed throughout the matrix, supporting the hypothesis that DHA or other organic components were successfully associated with the nanoparticle surface. Such embedding may improve colloidal stability and control release properties for biomedical applications. Overall, TEM analysis confirmed the successful synthesis of DHA-loaded AgNPs with predominantly spherical morphologies, nanoscale sizes, and surface coating or encapsulation by DHA. Despite minor aggregation at lower magnifications, the particles remained discrete and structurally stable, which is favorable for their potential biomedical and drug-delivery applications.

Microalgae are recognized as a sustainable and high-quality source of DHA, an omega-3 polyunsaturated fatty acid with established roles in cardiovascular, neurological, and metabolic diseases. Microalgae-based DHA-loaded AgNPs represent a cutting-edge intersection of sustainable biotechnology and advanced nanomedicine (Zhang et al., 2020; Sidorowicz et al., 2023; Ojha et al., 2025). As shown in Table 1, AgNPs are widely recognized for their broad-spectrum antimicrobial properties and unique physicochemical properties. The convergence of microalgal biotechnology and nanoparticle engineering, specifically the development of microalgae-based DHA-loaded AgNPs, offers a promising platform for multitargeted biomedical intervention. This report synthesizes the current knowledge on the therapeutic, diagnostic, and drug delivery applications of DHA/AgNPs, emphasizing green synthesis, biological interactions, and future research directions (Gnanakani et al., 2019; Singh et al., 2019; Bailey et al., 2025; Ugya et al., 2025). Nanoparticle carriers improve DHA solubility, stability, and bioavailability, thereby enhancing therapeutic outcomes in cardiovascular, neurological, and metabolic disorders (Puri et al., 2016; Jiang et al., 2024). However, challenges such as toxicity assessment, standardization, and economic scalability remain unresolved. Addressing these gaps through interdisciplinary research and process innovation is key to realizing the full potential of DHA/AgNPs in clinical and industrial settings (Gnanakani et al., 2019; Hussein et al., 2019; Ugya et al., 2025).

**TABLE 1:** Comparative biomedical applications of microalgae-based DHA/AgNPs.

Application	Mechanism/Pathway	Key advantages	Limitations/Challenges	Ref.
Diabetes Therapy	Membrane remodeling, insulin signaling, anti-inflammatory	Superior efficacy, targeted action	Long-term safety, dose optimization	Hussein et al. (2019), Das et al. (2025)
Antimicrobial	ROS generation, membrane damage, biofilm inhibition	Broad-spectrum, reduced toxicity	Standardization, resistance	da Cunha et al. (2023), Pereira et al. (2025)
Anticancer	ROS-mediated apoptosis, lipid peroxidation	Selective cytotoxicity, targeted delivery	Preclinical stage, cytotoxicity thresholds	Reynolds et al. (2014), Yang et al. (2018), Do et al. (2025)
Drug Delivery	Enhanced bioavailability, controlled release	Improved stability, absorption	Scale-up, formulation cost	Liu et al. (2021), Do et al. (2025)
Diagnostic Imaging	Optical properties	Non-invasive, real-time imaging	Clinical validation	Takahashi et al. (2017), Roome and Razzak (2020), Oloninefa et al. (2022)

## 4 Conclusion

This study demonstrates the successful stabilization of microalgae-derived DHA through its integration with AgNPs, yielding a DHA-loaded AgNP system with well-defined physicochemical characteristics and potential for future nanocarrier-based biomedical applications. Chemical and structural analyses verified the successful incorporation of DHA into AgNPs, suggesting enhanced stability and potential controlled-release behavior. Despite the demonstrated potential of DHA–AgNPs in biomedical applications, further investigation is required to refine the formulation strategies, assess their biocompatibility, and validate their *in vivo* therapeutic performance. These findings can be used to develop nanotechnology-based omega-3 fatty acid delivery systems that align with sustainable health trends (Moreira et al., 2022).

## Data Availability

The original contributions presented in the study are included in the article/supplementary material, further inquiries can be directed to the corresponding author.
